# Label-Free Quantitative Proteomics Analysis of Adriamycin Selected Multidrug Resistant Human Lung Cancer Cells

**DOI:** 10.3390/biom12101401

**Published:** 2022-10-01

**Authors:** Esen Efeoglu, Michael Henry, Martin Clynes, Paula Meleady

**Affiliations:** 1 National Institute for Cellular Biotechnology, Dublin City University, D09 NR58 Dublin, Ireland; 2 SSPC, The Science Foundation Ireland Research Centre for Pharmaceuticals, V94 T9PX Limerick, Ireland; 3 School of Biotechnology, Dublin City University, D09 E432 Dublin, Ireland

**Keywords:** drug resistance, DLKP, label-free quantitative proteomics, lipid metabolism, ABC transporters, response to drug

## Abstract

The development of drug resistance in lung cancer is a major clinical challenge, leading to a 5-year survival rate of only 18%. Therefore, unravelling the mechanisms of drug resistance and developing novel therapeutic strategies is of crucial importance. This study systematically explores the novel biomarkers of drug resistance using a lung cancer model (DLKP) with a series of drug-resistant variants. In-depth label-free quantitative mass spectrometry-based proteomics and gene ontology analysis shows that parental DLKP cells significantly differ from drug-resistant variants, and the cellular proteome changes even among the drug-resistant subpopulations. Overall, ABC transporter proteins and lipid metabolism were determined to play a significant role in the formation of drug resistance in DKLP cells. A series of membrane-related proteins such as HMOX1, TMB1, EPHX2 and NEU1 were identified to be correlated with levels of drug resistance in the DLKP subpopulations. The study also showed enrichment in biological processes and molecular functions such as drug metabolism, cellular response to the drug and drug binding. In gene ontology analysis, 18 proteins were determined to be positively or negatively correlated with resistance levels. Overall, 34 proteins which potentially have a therapeutic and diagnostic value were identified.

## 1. Introduction

Lung cancer is one of the most lethal solid malignancies causing more deaths than any other type of cancer worldwide. Depending on the type and location of the tumour, lung cancer is usually treated via surgery, chemotherapy, immunotherapy and radiotherapy. However, ~50% of the patients cannot benefit from these therapy regimens due to intrinsic or acquired drug resistance, leading to a 5-year survival rate of ~18% [1]. To date, various mechanisms such as drug transport and metabolism, involvement of cellular signalling pathways and genetic/epigenetic modifications have been shown to be involved in the development of resistance in cancer cells [2].

Among chemotherapeutic drugs, Adriamycin (Adr) has been widely used for the treatment of lung cancer due to its capability to inhibit topoisomerase activity, induce the generation of reactive oxygen species (ROS) and intercalate with DNA, ultimately altering cellular growth and proliferation. To combat the action of Adr, cells have been identified to develop defence mechanisms including increased expression of drug-efflux pumps to remove Adr from the cell [3], elevated levels of glutathione transferases to compensate for the ROS formation [4] and increased expression of antiapoptotic proteins to prevent cell death [5]. The role of drug-efflux pumps and multidrug resistance pumps such as ATP-binding cassette (ABC) transporters have been studied extensively and higher expression of ABC transporters has been reported to facilitate the removal of cytotoxic agents including Adr from the cancer cells, leading to the formation of resistance. A variety of these transmembrane proteins have been identified such as p-glycoprotein (MDR1, ABCB1), MDR-associated protein 1 (MRP1, ABCC1) and breast cancer resistance protein (BCRP, ABCG2). These membrane transporters effectively remove topoisomerase inhibitors, taxanes and antimetabolites from the cancer cells [3,6,7]. 

Administration of the chemotherapeutic agents triggers a cascade of events leading to an altered global proteome. Therefore, an in-depth proteomic investigation of the cellular proteome using advanced state-or-the-art mass spectrometry can provide an invaluable opportunity to enhance our understanding of resistance mechanisms as well as to identify biomarkers with prognostic and diagnostic potential. DLKP cells, which was established from metastasis of poorly differentiated squamous cell carcinoma of the lung in a male patient, provide a great platform to study drug resistance [8]. The DLKP cell line encompasses a variety of subpopulations which are shown to exhibit various levels of resistance to chemotherapeutic agents [8]. In terms of its drug-resistant subpopulations, DLKP-A was obtained by continuous exposure of DLKP cells to high levels of Adr, and drug-resistant subclones were isolated randomly from DLKP-A [6,9]. Overall 10 subclones with differing chemotherapeutic resistance were selected and 2 of these subclones DLKP-A2B and DLKP-A5F were employed in this study. In order to increase the level of drug resistance, DLKP-A cells were further exposed to increasing concentrations of Adr, and the selected subpopulation was established as DLKP-A10 [10].

In this study, the global proteome of the DLKP cell line and its Adr resistant subpopulations (DLKP-A2B, DLKP-A, DLKP-A5F and DLKP-A10) were investigated to identify differentially expressed proteomes. Subsequent gene ontology analysis aimed to establish a panel of novel biomarkers which can help to overcome drug resistance. Finally, identified biomarkers were compared to differential expression data obtained from a variety of lung cancer cells (SKMES-1 and COR23) for validation of the biomarkers in multiple lung cancers.

## 2. Methodology

### 2.1. Cell Culture

**Figure 1:**
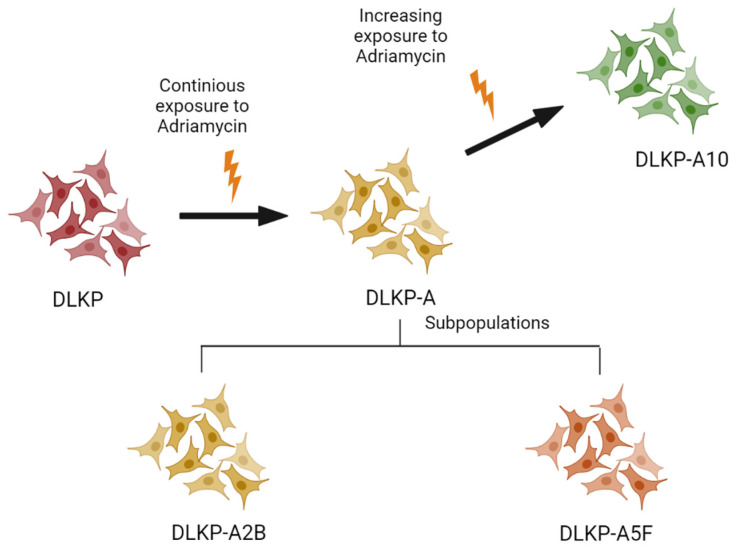
Establishment of drug-resistant cell lines. Resistance levels; pDLKP < DLKP-A2B < DLKP-A < DLKP-A5F < DLKP-A10.

DLKP cells, poorly differentiated squamous carcinoma, were established in the National Institute of Cellular Biotechnology (NICB), Dublin City University (DCU) along with the drug-resistant subpopulations, DLKP-A2B, DLKP-A, DLKP-A5F and DLKP-A10 (Figure 1) [6,9,10,11]. DLKP-A was established by continuous exposure of the DLKP cells (pDLKP) to escalating concentrations of Adr (Step 1: 0.05 µg/mL–0.8 µg/mL Adr for 11 months, Step 2: 0.8 µg/mL–1.75 µg/mL for 3 months and Step 3: 1.75 µg/mL–2.1 µg/mL for 3 months) [6]. Briefly, during these time periods, Adr was added at a concentration which allows 5% survival. When cells were adapted, appeared healthy and reached to confluence, drug concentration was doubled. DLKP-A cell line was observed to have a heterogeneous nature and this was further investigated by Heenan et al. which led to the isolation of drug resistant subclones [10]. Drug-resistant subclones, DLKP-A2B and DLKP-A5F, which are employed in this study, were isolated randomly from DLKP-A [6,9]. Briefly, DLKP-A cells were plated on 35 mm tissue culture dishes at the density of 50 cells per dish and grown in 5% CO_2_ at 37 °C for 24 h. Cells were observed under microscope for singular and dispersed adhesion on the surface and regularly fed with cloning medium (1:1 ratio of mixture of growth medium: conditioned medium (obtained from DLKP-A after 24 h, filtered through 0.2 micron filter and stored at 4 °C)) supplemented with 10% FCS and 1% L-Glutamine. When each single cell formed a colony compromises approximately 50 cells, colonies were separately sub-cultured using cloning rings. The selected colonies were scaled up using 96 well plates, 48 well plates, 24 well plates, 25 cm^2^ flask and 75 cm^2^ flasks, respectively. The clones were selected and routinely maintained in culture in the absence of Adr [10]. The DLKP-A10 variant was established via exposure of DLKP-A cells to increasing concentrations of Adr to a final concentration of 17.25 µM. Once DLKP-A10 was established, cells were cultured in 1:1 ratio of DMEM and DMEM-F12 supplemented with 5%FCS and 1% L-Glutamine in the absence of Adriamycin [11].

Toxicity determination of cytotoxic drug dilutions on the DLKP cells and drug-resistant variants has been carried out using the acid phosphatase assay. The plates were incubated with the cytotoxic drug dilutions for 7 days until control cells approached confluency. The IC50 was calculated by plotting the percent survival versus concentrations of the cytotoxic drug [6,7,9]. Drug-resistant subpopulations has been shown to exhibit cross-resistance to various chemotherapeutic reagents such as VP-16, colchicine, vincristine and cisplatin by Redmond et al. [6]. Drug-resistant subpopulations have been shown to be stable in culture for at least three months in the absence of drug [6,10] and therefore grown in Adriamycin free medium for proteome comparison with parental DLKP cell line.

For collection of samples for proteomic investigation, all DLKP cells were grown in DMEM-F12 medium supplemented with 5% FBS and 1% L-glutamine. Cells were sub-cultured via trypsinisation every 3–4 days when they reached the confluence of approximately 70–80%. All cell lines were mycoplasma tested regularly throughout the study. In order to obtain samples for mass spectrometry analysis, cells were removed by trypsinisation and pelleted via centrifugation. Pellets were washed three times with PBS to remove any cell culture medium residues, snap-frozen using liquid nitrogen and stored at −80 °C prior to Mass Spectrometry (MS) sample preparation. Three pellets were obtained from three independent subcultures of each cell line for whole-cell proteome analysis (3 × 5 cell lines; in total, 15 cell pellets are collected, compared and analysed). 

### 2.2. Preparation of Mass Spectrometry Samples

The sample preparation for MS was carried out using the PreOmics iST 96x kit by following the manufacturer’s instructions (PreOmics GmbH, Martinsried, Germany). The kit contains all the required solutions (Lyse buffer, digestion enzyme mix, wash solutions and elution solutions) to extract and digest proteins present in biological samples such as cells and tissues. The PreOmics iST kit requires a maximum of 100 µg of protein sample as a start material. Protein concentrations of cell pellets were determined using a BCA assay kit (Thermo Fisher Scientific, Dublin, Ireland) and volumes containing 100 µg of protein were transferred into 1.5 mL tubes for lysis, reduction and alkylation. A total of 50 µL of LYSE solution was added to the 100 µg protein samples and incubated for 10 min at 95 °C with continuous mixing (1000 rpm). Samples were allowed to cool and then transferred to cartridges. A total of 210 µL of RESUSPEND solution was added into each DIGEST tube and the samples were shaken at 500 rpm at room temperature for 10 min to dissolve the digestion enzyme. A total of 50 µL of the DIGEST was added into cartridges and protein samples were digested for 2 h at 37 °C with continuous mixing (500 rpm). Following 2 h of digestion, STOP solution was added to the cartridges and samples are shaken (500 rpm) at room temperature for 1 min to stop the digestion. The cartridges were spun in a microcentrifuge at 3800 rcf for 3 min to remove the DIGEST solution. WASH1 and WASH2 solutions were used to stack the digested peptides in the cartridge filter. After each wash, the cartridge was spun at 3800 rcf for 3 min and flowthrough was discarded. Digested peptides were collected into a fresh tube using 50 µL of ELUTE solution, placed in a SpeedVac (45 °C), and spun until completely dry. Digested peptides were resuspended using LC-LOAD solution at 1 g/L and sonicated for 5 min. 

In order to observe the presence of biomarkers in various cell lines, the DE data obtained from Lung squamous cell carcinoma (SK-MES-1) versus its drug resistant variant and Non-Small Cell Lung Cancer (COR23) versus its drug resistant variant were used. It is important to note that these samples were collected as frozen cell pellets and prepared using the PreOmics iST kit by following the manufacturer’s instructions prior to MS data collection, data analysis and gene ontology analysis. Three pellets were obtained from three independent subcultures of each cell line for whole-cell proteome analysis.

### 2.3. Mass Spectrometry (the Nano LC-MS/MS)

The nano LC-MS/MS analysis and profiling of digested protein samples was carried out using a Dionex Ultimate 3000 Reversed-phase Capillary high-pressure Liquid Chromatography (RSCLC) nano system coupled to a hybrid linear Iontrap/Orbitrap Fusion Tribrid mass spectrometer (Thermo Scientific, Dublin, Ireland) throughout the study. PepMap100 (C18, 300 µm × 5 mm) and Acclaim PepMap 100 (5 µm × 50 cm, 3 µm bead diameter) columns were used as trapping and analytical column, respectively. One microgram from each sample was loaded onto the trapping column at a flow rate of 25 µL/min with 2% (*v*/*v*) acetonitrile (ACN) and 0.1% (*v*/*v*) trifluoroacetic acid (TFA) for 3 min. Samples were resolved in the analytical column using a binary gradient of formic acid solution (0.1% (*v*/*v*) formic acid in LC-MS grade water) and ACN/formic acid solution (80% (*v*/*v*) ACN, 0.08% (*v*/*v*) formic acid in LC-MS grade water). A flow rate of 300 nL/min was used to elute peptides. Peptide ionization was carried out at 320 °C and 2.0 kV. The data-dependent acquisition was carried out using a full scan range of 380–1500 *m*/*z* and each sample was run for 2 h. MS/MS scan conditions were set as follows: a targeted AGC value of 2 × 10^4^ and a maximum fill time of 35 ms. The number of selected precursor ions for fragmentation was determined using a top-speed acquisition algorithm and ions were isolated in the quadrupole using an isolation width of 1.6 Da. Dynamic exclusion was applied to analysed peptides after 60 s and peptides with a charge state between 2+ and 7+ were analysed. Fragmentation of precursor ions was carried out using higher energy collision-induced dissociation and the resulting MS/MS ions were detected via a linear ion trap. 

### 2.4. Differential Expression (DE) Analysis

The raw LC-MS/MS files were transferred and analysed using Proteome Discoverer version 2.1 (Thermo Scientific, Dublin, Ireland) and Progenesis QI for Proteomics (NonLinear Dynamics, Waters, Newcastle, UK). Proteome Discoverer software allowed the identification of proteins/peptides by using the SEQUEST HT algorithm and searched the identified peptides against the Uniprot human database (fasta database downloaded in January 2021). A total of 20 ppm precursor mass tolerance and 0.6 Da fragment mass tolerance were used in all searches in Proteome Discoverer. Oxidation of methionine was set as a dynamic modification and carbamidomethylation of cysteine was set as a static modification. A maximum of two missed cleavages was allowed during the search. A false discovery rate was set to be less than 5% using Percolator. Trypsin was set as the digestion enzyme for all samples. 

Differential expression (DE) analysis was carried out using Progenesis QI for Proteomics to determine the cellular proteome involved in the formation of drug resistance. The lists of differentially expressed proteins were filtered by ANOVA < 0.05 and at least a fold change of ±1.5. The Gene Ontology (GO) analysis of differentially expressed proteins was used to identify significantly enriched KEGG pathways, biological processes and molecular functions which play a role in the formation of resistance. 

### 2.5. Gene Ontology

STRING gene ontology (https://version-11-0.string-db.org/, accessed on 1 June 2021) and PANTHER gene ontology (http://www.pantherdb.org/, accessed on 1 June 2021) were used to determine enriched biological processes, molecular functions, and KEGG pathways, as well as protein–protein interactions. Strength and False Discovery Rate (FDR) were used to determine the most significant enrichments. Strength describes how large the enrichment is and it is calculated using Log_10_ (observed/expected). Observed relates to the number of proteins in the network that were annotated with a term and expected relates to the number of proteins that we expect to be annotated with this term in a random network of the same size. FDR describes how significant the enrichment is, and *p*-values are corrected for multiple testing within each category using the Benjamini–Hochberg procedure [12].

### 2.6. Western Blot for Validation

Western blotting was performed on cell lysates obtained from DLKP cells and drug resistant variants. Samples were separated using a Bolt™ 4 to 12%, Bis-Tris, 1.0 mm, Mini Protein Gels (Thermo Fisher Scientific, Dublin, Ireland) with 20 µg loaded per well. After Western blotting, blots with primary antibodies (ABCB1 (MDR1), FSCN1, LIMA1 and β-Actin) were incubated overnight at 4 °C (Cell Signalling Technologies, Dublin, Ireland). The IRDye^®^ 800CW Donkey anti-Rabbit IgG and IRDye^®^ 800CW Goat anti-Mouse IgG were used as secondary antibodies (Li-Cor BioSciences, Lincoln, NE, USA). Signal from secondary antibodies was measured using Li-Cor Odyssey imaging system at 800 nm.

## 3. Results and Discussion

### 3.1. Drug Resistance in DLKP Subclones and the Role of ABC Transporters: ABCB1 and ABCC1

**Figure 2:**
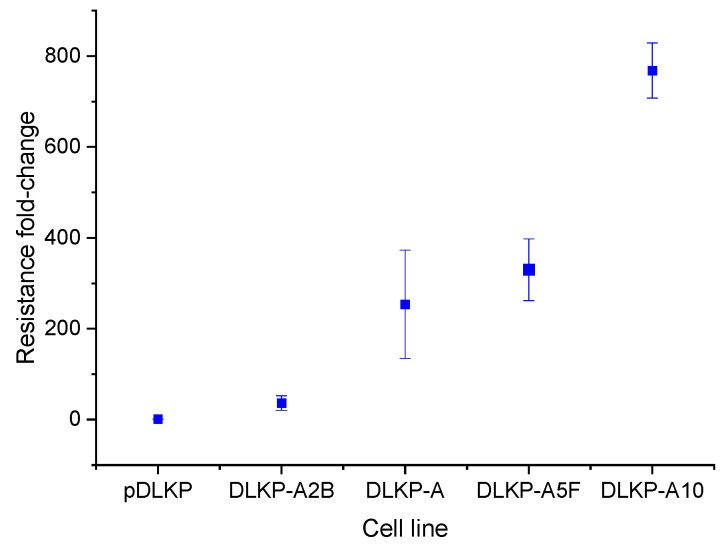
Drug resistance and profile of DLKP and DLKP subpopulations. Fold resistance is calculated as Fold Resistance = IC50_(subpopulation cell line)_/IC50_(DLKP-parental cells)_. ±SD is obtained from at least 3 independent experiments. The figure is adapted from Keenan et al. [9].

Tumour heterogeneity originating from tumour subtypes and mutations on the same subtype fuels drug resistance and results in differing responses to therapy [13,14]. The DLKP cell line encompasses ten different subpopulations which are shown to exhibit various levels of resistance to chemotherapeutic agents [10]. This variety originating from a single cell line makes DLKP a highly attractive candidate to study the formation of resistance. Previously, the varying drug sensitivities of parental DLKP (pDLKP) and its subclones have been demonstrated by Keenan et al. [9]. In this study, DLKP subpopulations which exhibit low, medium and high resistance to Adr were selected to investigate variances in cellular proteome leading to chemoresistance by using advanced MS-based proteomics. Selected clones: DLKP-A2B, DLKP-A, DLKP-A5F and DLKP-A10 show 36.5, 254, 330 and 768-fold higher resistances to Adr compared pDLKP, respectively (Figure 2). 

Drug resistance formation in various cell lines has been widely related to ABC transporters, which provide the efflux of cytotoxic reagents from cells in the presence of ATP, ultimately decreasing the drug presence [15,16,17]. To date, 49 ABC transporter proteins have been identified and some of these have been implicated in the formation of drug resistance [15,18,19]. Among ABC transporters, ABCB1 (also known as p-glycoprotein and MDR1) was the first identified and is expressed in various organs such as the gut, liver, kidney, intestines and physiological barriers [20]. Its role in drug resistance against a wide variety of chemotherapeutic agents such as paclitaxel and tyrosine kinase inhibitors has been reported [21,22,23]. The accumulated knowledge on the function of ABCB1 in the development of drug resistance drove the creation of novel therapeutic strategies using ABCB1 inhibitors and substrate-drugs to re-sensitise drug-resistant cells. A variety of inhibitors of multi-drug resistance protein have been shown to reduce levels of drug resistance in all drug-resistant variants of DLKP cells [6,10,11]. Furthermore, the investigation of new drug candidates against the ABCB1 transporter was recommended by the Food and Drug Administration (FDA) [24]. Clynes et al. showed the expression of ABCB1 in pDLKP cells and drug-resistant DLKP-A cells using immunofluorescence and Western blotting which indicated the overexpression of p-glycoprotein, and its direct role in the formation of drug resistance was shown using a specific antisense oligonucleotide [6]. Heenan et al. further investigated the expression of ABCB1 in DLKP subclones using RNA analysis and Western blotting to determine the possible interactions between different biochemical/genetic mechanisms and drug resistance levels. Various ABCB1 expression profiles of the subclones were shown to be linked to several mechanisms, including overexpression of ABCB1 and varying levels of topoisomerase II [10]. 

**Figure 3:**
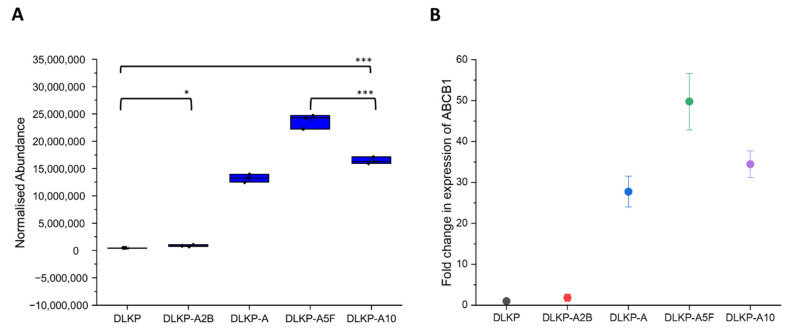
Expression levels of ABCB1 in pDLKP cell line and drug-resistant subpopulations. (**A**) Normalised abundance of ABCB1 obtained from DE analysis of pDLKP cell line and drug-resistant subpopulations. For each cell line, data is obtained from 3 independent samples and one-way ANOVA is applied to show statistical significance. * and *** represents *p* < 0.05 and *p* < 0.001, respectively. (**B**) Fold-change of ABCB1 expression in pDLKP cell line and drug-resistant subpopulations. The pDLKP cell line is set to 1 for comparison of drug-resistant subpopulations. Each subpopulation is indicated with colors as follows; DLKPA2B (red), DLKP-A (blue), DLKP-A5F (green) and DLKP-A10 (purple). Data are shown as fold-change ± standard deviation from three independent experiments.

In this study, an in-depth label-free MS-based proteomic analysis of the DLKP cell line and its drug-resistant subpopulations also showed the presence of ABCB1, which was detected by MS/MS with 47 unique peptides and a *p*-value of 3.55 × 10^−13^. DLKP-A2B showed minimal difference compared to parental cells for the expression of ABCB1 (1.8-fold, *p* = 0.05), however expression levels increase dramatically for other resistant subpopulations with fold changes of up to 50 (Figure 3A,B and Supplementary Table S1). It is important to note that although the resistance level of DLKP-A10 was higher than DLKP-A5F, ABCB1 expression was observed to be relatively low. This can be attributed to the involvement of multiple mechanisms contributing to the drug resistance levels, especially for the cells which exhibit high levels of resistance.

ABCC1, also known as MRP1, was the second ABC transporter identified and is associated with the development of drug resistance by glutathione-mediated cellular efflux of chemotherapeutic drugs, leading to poor drug sensitivity [25]. Its overexpression was previously shown to affect resistance to doxorubicin, etoposide and vincristine. Although ABCB1 and ABCC1 show similar resistance profiles, they exhibit different substrate selectivity. ABCB1 is involved in the transport of lipophilic molecules with a slight positive charge, whereas ABCC1 can transport lipophilic anions and shows specificity to a broad range of substrates such as glutathione conjugates, sulphate conjugates, anticancer drugs, metals, organic anions and lipid analogues [26,27,28]. In this study, ABCC1 was significantly upregulated in DLKP-A (18.8-fold) and DLKP-A10 (5.8-fold), but was not expressed in DLKP-A2B and DLKP-A5F, making the fold change infinite (Supplementary Table S1). However, no correlation was observed between increasing levels of resistance of the DLKP subpopulations and expression levels of ABCC1.

### 3.2. Interaction of ABCD1 and ABCD3-Transporter Proteins with Fatty Acid Metabolism and Peroxisome Pathway in Drug Resistance

In addition to ABC transporters which have established roles in drug resistance such as ABCB1 and ABCC1, some other ABC transporters were identified in DLKP subpopulations such as ATP-binding cassette sub-family D member 1 (ABCD1) and ATP-binding cassette sub-family D member 3 (ABCD3) (Supplementary Table S1). ABCD1 (ALDP) and ABCD3 (PMP70) are involved in the regulation of very long-chain fatty acid (VLCFA) transport on the peroxisome membrane [29,30]. Fatty acids are essential to various metabolic processes during cancer development such as sustaining the membrane biosynthesis for rapid proliferation and providing an energy source during metabolic stress [31]. Although they are known to have oncogenic roles, their mechanism of action and role in drug resistance are not completely understood. Recently, the accumulation of VLCFAs in triacylglycerol and nonesterified forms have been shown in colorectal cancer by Hama et al. using a lipidomics approach [32]. Very long-chain acyl-CoA synthetase 3 (ACSVL3) knockdown was shown to result in reduced colony formation and HEPG2 growth rates were shown to decrease by 65–76% [33]. Hillebrand et al. studied ABCD1 dysfunction and its consequences on the fatty acid synthesis-transport system and showed the binary interactions of ABCD1 with ACYL, FASN and ACC [34]. 

**Figure 4:**
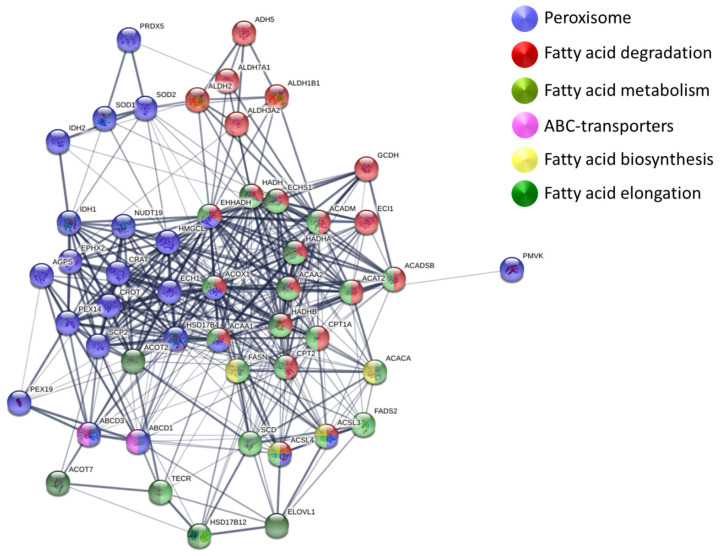
Network of proteins involved in fatty acid metabolism and their interaction with ABC-transporters. Proteins related to various biological processes and protein groups are colour coded as follows: Peroxisome related proteins and ABC-transporters are indicated with purple and pink, respectively. Proteins related to biological processes such as Fatty acid degradation, fatty acid metabolism, fatty acid biosynthesis and fatty acid elongation are indicated with red, green, yellow and dark green, respectively. Protein–protein interactions are shown via grey lines with thickness of the band indicating different strengths of the data support (experiments, data mining, neighbourhood co-occurrence are included to contribute confidence) identified by Gene Ontology analysis. The confidence strength was medium (0.400). (Full DE list and expression details, Supplementary File S1).

In our study, DE analysis of pDLKP cells and drug-resistant subpopulations showed significant enrichments in KEGG pathways such as fatty acid metabolism, fatty acid degradation, fatty acid elongation, biosynthesis of unsaturated fatty acids, fatty acid biosynthesis and peroxisome (Figure 4, Supplementary File S1-Section A). A total of 50 proteins including ABCD1 and ABCD3, contributed to enrichment in these pathways with 21 of them being downregulated and 13 of them being upregulated in drug-resistant subpopulations. The highest and lowest expression levels of the remaining 14 proteins were observed within drug-resistant subpopulations, e.g., ACAA2 being expressed highest in A2B and lowest in A10 (Supplementary File S1-Section B). 

β-oxidation of fatty acids can occur both in mitochondria and peroxisomes. Carnitine plays a significant role in the oxidation of long-chain fatty acids, energy production and transport of the toxic compounds out of the mitochondria preventing their accumulation [35,36]. A fatty acid is taken into the mitochondria by carnitine palmitoyltransferase 1 (CPT1) and carnitine palmitoyltransferase 2 (CPT2). CPT1 is an enzyme located in the outer mitochondrial membrane and it binds fatty acid to the carnitine shuttle, then CPT2 detaches the fatty acid once it passes into the inner mitochondrial membrane. In the mitochondria, degradation comprises 4 steps: oxidation, hydration, 2nd oxidation and thiolation, removing 2 atoms of carbon in the form of Acetyl-CoA. Then, Acetyl-CoA feeds into the citrate cycle or many anabolic pathways depending on the cell state. For the fatty acid degradation pathway, 11 proteins including CPT1 were found to be downregulated in drug resistant cells, whereas only 8 proteins were upregulated. All 8 upregulated proteins were observed to be involved in the 1st oxidation reaction, the following downstream enzymes which are involved in hydration, 2nd oxidation and thiolation were downregulated. This suggests the downregulation of the mitochondrial β-oxidation (degradation) pathway of fatty acids in drug-resistant cells. Among downregulated proteins, Aldehyde dehydrogenase (ALDH2), Enoyl-CoA hydratase (ECHS1), 3-ketoacyl-CoA thiolase (ACAA1), Acetyl-CoA acetyltransferase (ACAT2) and Carnitine O-palmitoyltransferase 1 (CPT1) showed the most significant fold changes and they decreased in drug-resistant cells by 14.3, 3.3, 2.7, 2.6 and 2.6-fold, respectively. ECHS1, ACAA1, ACAT2 and CPT1 are involved in the β-oxidation of fatty acids, whereas ALDH2 is known to be a major enzyme of α-oxidation of branched-chain fatty acids in peroxisomes. Among upregulated proteins, Aldehyde dehydrogenase X (ALDH1B1), Aldehyde dehydrogenase family 3 member A2 (ALDH3A2) and Glutaryl-CoA dehydrogenase (GCDH) were upregulated by 2.6, 2.6 and 4.4-fold, respectively, and are associated with the production of fatty acids. Fatty acid synthase (FASN) and Acetyl-CoA carboxylase 1 (ACACA) were also upregulated in several drug-resistant subpopulations. FASN is involved in the production of fatty acids and ACACA produces Malonyl-CoA which is used by FASN to produce fatty acids. FASN expression is higher in cancer cells compared to healthy counterparts of the same tissue [37,38] and is associated with a poor prognosis for a wide range of cancers (including lung cancer). In recent years, cellular lipids and lipid metabolism garnered significant attention in cancer research due to their association with various metabolic processes which provide necessary building blocks of tumour sustainability. In addition to this, they provide an alternative fuel source for ATP synthesis ultimately escalating tumour growth and sustainability, especially in tumours with lipogenic phenotypes [39,40]. Our findings from the comparison of drug-resistant cells and subsequent gene ontology analysis further suggest the association of lipid metabolism with significantly higher levels of resistance. 

Fatty acid synthesis and breakdown are known to be competing pathways, which correlates with the results shown above. Inhibition of FASN has been shown to delay disease progression in a xenograft model of ovarian cancer [41], which shows its potential to be used as a biomarker. The cancer cells can be re-sensitised to chemotherapeutic agents by promoting fatty acid breakdown. Therefore, proteins related to fatty acid synthesis such as ALDH1B1, ALDH3A2, GCDH, FASN and ACACA, which were identified to be differentially expressed in our study, can potentially be used as a biomarker of drug resistance in lung cancer cells.

As seen in Figure 4, 24 peroxisome-related proteins which interact with ABC-transporters (indicated in blue) were identified to be upregulated or downregulated in drug-resistant DLKP subpopulations, with 16 of them having either their highest or lowest expression in pDLKP cells. The other 8 did not follow a trend between parental and drug-resistant cell lines. Peroxisomes play an essential role in redox signalling and lipid homeostasis in cells by contributing to a variety of cellular mechanisms such as fatty acid oxidation, biosynthesis of lipids and detoxification. Import of fatty acids into peroxisomes requires recognition by early peroxins, PEX3, PEX16 and PEX19. In our study, Peroxisomal biogenesis factor 19 (PEK19) was observed to be downregulated by 2.7-fold in drug-resistant cells. Nine other peroxisomal proteins related to recognition mechanism, α-oxidation of fatty acids and unsaturated fatty acid β-oxidation such as ALDH2 (14.3-fold) and ECH1 (2-fold) were also downregulated. DE analysis of pDLKP and drug-resistant subpopulations showed significant upregulation of Peroxisomal carnitine O-octanoyl transferase (CROT) and Bifunctional epoxide hydrolase 2 (EPHX2). Although EPHX2 is significantly upregulated in drug-resistant cell populations, it is ubiquitously produced by many different cell types, limiting its potential in targeted therapies. CROT regulates the breakdown of VLCFA and its expression increased by 52.8-fold in drug resistance cells. In addition to its association with the fatty acid breakdown and high fold change, the function of CROT in cancer and drug resistance remains to be discovered. Therefore, CROT could potentially be a valid biomarker. 

Overall, downregulation of ABCD1 can be related to significant enrichments in lipid metabolic pathways, leading to the downregulation of fatty acid breakdown and upregulation of fatty acid synthesis. ABCD1 inhibits the transfer of VLCFA into mitochondria and peroxisomes by blocking their recognition and uptake, as well as downregulation of proteins involved in downstream processes in drug-resistant cells. 

### 3.3. Other ABC-Transporters and Their Expression Profiles in Drug-Resistant DLKP Subpopulations

The ABCE and ABCF subfamily of ABC transporters were also identified to be differentially expressed in drug resistant variants by 2.2 and 1.9-fold, respectively (Supplementary Table S1). This subfamily of proteins is known to have two nucleotide-binding proteins; however, their transmembrane domains have not yet been identified. Therefore, their association with membrane transport is not clear. The ABCF2 protein, an Nrf2 target gene, has been shown to contribute to cisplatin resistance in ovarian cancer cells [42]. Another study showed that the knockdown of ABCE1 affects the sensitivity of A549 cells against certain chemotherapeutic agents [43]. Although differential expression of these proteins resulted in very low fold-changes, their presence in DLKP cells suggests their possible association with drug resistance and can be further investigated to determine their relation to poor drug sensitivity. 

### 3.4. Expression Levels of Plasma Membrane-Related Proteins in pDLKP and Its Drug-Resistant Subpopulations

**Figure 5:**
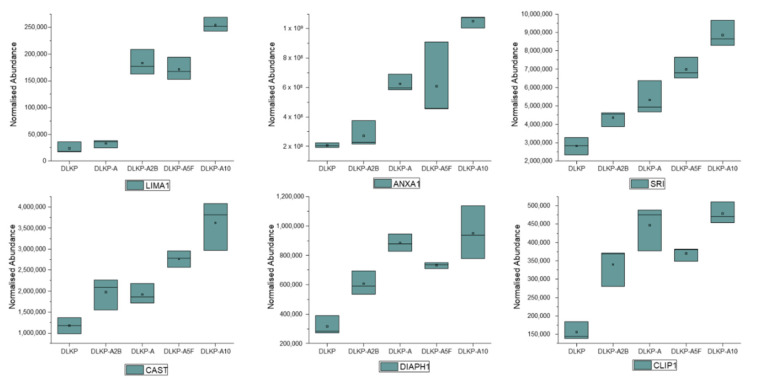
Expression profiles of upregulated membrane proteins in pDLKP and its drug-resistant subpopulations. For DE analysis 3 independent samples per cell line were analysed and normalised abundances of proteins of membrane proteins plotted against cell variant types. LIMA1 (LIM domain and actin-binding protein 1), ANXA1 (Annexin A1), SRI (Sorcin), CAST (Calpastatin), DIAPH1 (Protein Diaphanous Homolog 1), CLIP1 (CAP-Gly domain-containing Linker Protein 1).

The UniProt (https://www.uniprot.org/, accessed on 1 January 2021) search of differentially expressed proteins (unique peptide number > 1 and max-fold change ≥ 2.5) showed that expression levels of 108 membrane proteins significantly altered between pDLKP cells and its drug-resistant subpopulations (Supplementary File S2). Among these 108 membrane proteins, 6 of them are found to be expressed lowest in pDLKP and their expression levels increased as a function of the resistance levels of the cells (Figure 5 and Supplementary File S2). In addition to these 6 proteins, Receptor-type Tyrosine-Protein Phosphatase C (PTPRC) was not detected in pDLKPs but was expressed in all drug-resistant subpopulations. However, expression levels of PTPRC did not correlate with the increase in resistance (Supplementary Figure S1A). LRBA was also observed to be expressed higher in drug-resistant subpopulations, however, no significant correlation was observed with the increase in resistance (Supplementary Figure S1B).

LIM domain and actin-binding protein 1 (LIMA1/EPLIN) plays a key role in the cholesterol mechanism as well as regulation of cytoskeleton and formation of epithelial cell junctions leading to metastasis via increased migration and invasion. The role of LIMA1 in the development of various cancer types such as breast, prostate and lung has been demonstrated and expression levels have been shown to reduce as cancer progresses [44]. Furthermore, the therapeutic potential of LIMA1 was investigated by manipulation of expression levels which led to reduced cell growth and motility [44,45]. However, our study suggests the presence of an alternative mechanism and role of LIMA1 for the development of resistance, since it was significantly upregulated in drug resistant cells (10.7-fold in DLKP-A10). 

ANXA1 is a Ca^2+^ dependent phospholipid linked protein which is commonly associated with cancer drug resistance to a variety of chemotherapeutic agents such as 5-fluorouracil, Adriamycin and etoposide via regulation of drug efflux systems such as ABCB1 [46,47]. Its role in the development of anti-inflammatory effects, Epithelial-Mesenchymal Transition (EMT), regulation of proliferation and apoptosis have also been shown in various cancer types [9,46,47,48]. Although the role of ANXA1 can vary depending on the cancer type, Belvedere et al. have shown that ANXA1 plays a role in maintaining an aggressive phenotype in knockout MIA PaCa-2 cells [49], which also correlates with our findings (increase from 1.3 to 5.1 fold in drug resistant variants).

Sorcin (SRI) is a commonly expressed protein in many tissues and plays a critical role in calcium-binding. Recently, its role in the development of cancer in various cancer types and correlation between the expression levels of SRI and ABCB1 have been shown. Knockdown of SRI leads to re-sensitisation of various cell types to chemotherapeutic agents [50]. In our study, SRI expression significantly increased with increasing levels of resistance (3.4-fold in DLKP-A10) and also correlated with the expression levels of ABCB1.

Calpastatin (CAST) is a natural inhibitor of calpains, which is released into the cytoplasm upon calcium influx and blocks calpain’s active sites. Calpains have been associated with a wide range of cellular processes from cellular survival, proliferation to migration and invasion, suggesting calpains and their endogenous inhibitor CAST, as promising therapeutic target [51]. The DE analysis showed the expression of CAST is increased gradually with increasing resistance, reaching highest of all in DLKP-A10 (3.1-fold). Even though the changes were not as prominent as CAST expression levels, Calpain-2 was downregulated in drug resistant subpopulations (Supplementary Figure S2). 

Recently, Protein Diaphanous Homolog 1 (DIAPH1) was classified as a cancer oncogene. DIAPH1 promotes myofibroblastic activation via regulation of Rab5a activity and TGFβ receptor endocytosis, and its knockout lead to limited tumour growth in mouse models [52]. A role for DIAPH1 was demonstrated in colon cancer via regulation of chromosomal instability during meta- and anaphase, a cancer hallmark [53]. Even though DIAPH1 gained more attention in cancer research in recent years, its association with drug resistance is unclear. In this study, expression of DIAPH1 increased in drug resistant cells corresponding to their level of resistance with the exception of DLKP-A5F (Figure 5, Supplementary File S3). 

CAP-Gly domain-containing Linker Protein 1 (CLIP1) binds to microtubules and plays role in the regulation of microtubule growth and bundling. CLIP1 has been associated with cancer development and drug resistance (imatinib and lorlatinib) in non-small cell lung cancer and gastric cancer, and selective depletion of a novel truncated variant of CLIP1 has been shown to sensitise resistant gastric cancer cells to imatinib [54,55]. Our results further suggests its expression levels are positively correlated (increasing from 2.2-fold to 3.1-fold) with resistance.

**Figure 6:**
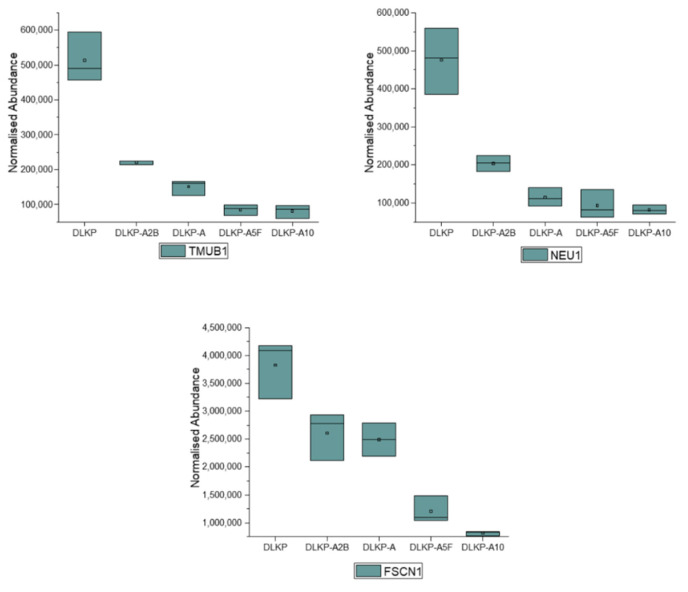
Expression profiles of downregulated membrane proteins in pDLKP and its drug-resistant subpopulations. For DE analysis 3 independent samples per cell line were analysed and normalised abundances of proteins of membrane proteins plotted against cell variant types. TMUB1 (Ubiquitin Like Domain Containing 1), NEU1 (Sialidase-1), FSCN1 (Fascin Actin-Bundling Protein 1).

11 of 108 proteins were observed to be significantly downregulated in drug-resistant subpopulations (Supplementary File S2). Expression levels of 3 of these proteins, Transmembrane Additionally, Ubiquitin Like Domain Containing 1 (TMUB1), Sialidase-1 (NEU1) and Fascin Actin-Bundling Protein 1 (FSCN1) inversely correlated with increasing levels of resistance (Figure 6). In addition to these proteins, Ribosomal Protein S15 (RPS15), Beta-2-Microglobulin (B2M), Interferon-Induced Transmembrane Protein 3 (IFITM3) and Mitogen-Activated Protein Kinase 1 (MAPK1) show inverse correlation with the resistance profile of the subpopulations, however they differ in their expression in DLKP-A5F (Figure 2). AP-1 complex subunit beta-1 (AP1B1), Integrin beta-1 (ITGB1), MHC class I polypeptide-related sequence B (MICB) and Magnesium transporter protein 1 (MAGT1) were also significantly downregulated in drug resistant subpopulations but no correlation with the level of resistance is observed (Supplementary Figure S4).

TMUB1, which also known as Hepatocyte Odd Protein Shuttling (HOPS) protein was downregulated by 2.3, 2.4, 6 and 6.3-fold for DLKP-A2B, DLKP-A DLKP-A5F and DLKP-A10, respectively, showing negative correlation with increasing levels of resistance. Although TMUB1-cancer drug resistance relationship has yet not been studied, its association with negative regulation of hepatocyte cell cycle and induction of apoptosis via interaction with p53 have been shown. [56,57]. Fu et al. further showed that the overexpression of TMUB1 leads to significant increases in the apoptosis of Hep3B cells whereas knockdown reduces the apoptosis rate [58]. 

Sialidases are involved in cleavage of sialic acids from glycans and play a part in cancer plasticity. NEU1, a sialidase precursor, gained attention due to its potential links with tyrosine kinase inhibitors and Toll-like receptors, and has emerged as a novel target for regulation of tumorigenesis [59]. This study further suggests the association of NEU1 with drug resistance by being highly downregulated on the plasma membrane of drug resistant subpopulations. The expression levels of NEU1 reduced 2.3, 4.2, 5.1 and 5.8-fold in DLKP-A2B, DLKP-A, DLKP-A5F and DLKP-A10, respectively. 

FSCN1 was significantly downregulated in drug resistant populations of DLKP cells, being lowest of all in the most drug-resistant line DLKP A10 (4.7-fold). FSCN1 has been shown to play a role in the organisation of actin filaments and formation of membrane ruffles and stress fibres, ultimately affecting cell motility and migration [60].

### 3.5. Enriched Pathways and Cellular Proteome Related to Enriched Drug-Resistance Mechanisms Determined by Gene Ontology (GO) Analysis

DE analysis of pDLKP cells with its drug resistant subpopulations showed upregulation and downregulation of 1266 proteins (Supplementary File S3). A total of 206 of these proteins were determined to contribute to the enrichment of biological processes such as drug metabolic processes, response to drug, regulation of cellular response to drug, negative regulation of response to drug and cellular response to drug. A total of 226 of 1266 are also shown to be involved in the enrichment of drug binding molecular function (Table 1).

9 proteins involved in the enrichment of drug related biological processes show a strong correlation with resistance levels. (Table 2 and Supplementary File S3, Supplementary Figure S4). Among these 9 proteins, Heme Oxygenase 1 (HMOX1) shows the most significant downregulation in drug resistant subpopulations and expressed levels of HMOX1 reduced by 56.1-fold in DLKP-A10 compared to pDLKPs. Although its role in DLKP cells has not been shown previously, the role of HMOX1 on development of chemoresistance has been found in some tumours [61,62]. In addition to HMOX1, Nucleobindin 1 (NUCB1), Cytochrome C Oxidase Subunit 4I1 (COX4I1) and FSCN1 were downregulated in drug resistant subpopulations (Table 2). HMOX1, COX4I1 and NUCB1 contribute to the enrichment of biological processes such as drug metabolic process and cellular response to drugs, whereas FSCN1 contributed to drug binding. 

As part of non-receptor guanine nucleotide exchange factors, a 63kDa Ca^2+^ binding protein, NUCB1 is involved in modulation of unfolded protein response via inhibition of ATF6 which leads to apoptosis and has been shown to fuel metastasis [63,64]. In pancreatic cancer cells, overexpression of NUCB1 has been shown to block autophagy upon gemcitabine exposure [64]. Although studies on COX4I1 are limited in terms of its role in drug resistance, it was shown to have a potential role in development of drug resistance in glioma cells through regulation of polycomb group complex 1 (BMI1) expression [65]. However, it is important to note that changes in expression levels of BMI1 was not observed in DLKP cells. 

6 proteins; Bifunctional epoxide hydrolase 2 (EPHX2), Ubiquinol-Cytochrome C Reductase Core Protein 2 (UQCRC2), Dihydrolipoamide S-Succinyltransferase (DLST), Succinate-CoA Ligase GDP-Forming Subunit Beta (SUCLG2), Pyruvate Dehydrogenase E1 Subunit Alpha 1 (PDHA1) and X-Ray Repair Cross Complementing 5 (XRCC5) are determined to contribute enrichment drug-related biological processes. Seven proteins, Heat Shock Protein Family A (Hsp70) Member 4 (HSPA4), Leucyl-TRNA Synthetase 1 (LARS1), Heat Shock Protein Family A (Hsp70) Member 4 Like (HSPA4L), DExH-Box Helicase 9 (DHX9), Chaperonin Containing TCP1 Subunit 4 (CCT4), Glycogen Phosphorylase B (PYGB) and Sedoheptulokinase (SHPK) contributed to enrichment of drug-binding (Table 2 and Supplementary File S3). EPHX2 expression significantly increased in DLKP drug resistant subpopulations and this increase highly correlated with the drug resistance levels of the subpopulations (Table 2). EPHX2 consists of two domains which encode soluble epoxide hydrolase as protein product. Soluble epoxide hydrolases involves in lipid phosphate hydrolysis. Although studies on EPHX2 in cancer lipid metabolism are limited, considering the significant regulation of lipid metabolism in DLKP drug resistant cells, overexpression of it can be attributed to presence of this connection in DLKP cells. 

PDHA1, as a subunit of Pryruvate Dehydrogenase, plays role in glucose metabolism by providing oxidative decarboxylation of pyruvate to produce acetyl-CoA. Due to its role in glycolysis and TCA cycle, it involves in cancer survival. In addition to glucose metabolism in cancer cells, Chen et al. has showed its interaction with lipid biosynthesis in prostate cancer [66]. PDHA1 has also been shown to contribute drug resistance of A549 cells and Human Esophageal Squamous Cancer Cells [67,68]. In DLKP cells, expression levels of PHDA1 increased in correlation with resistance level of the cells.

Succinate-CoA Ligase GDP-forming beta subunit (SUCLG2) regulates succinate metabolism and is involved in the conversion of succinyl-CoA to succinate and acetoacetyl-CoA (coupled with the substrate level phosphorylation of GDP to GTP) as part of the TCA cycle. SUCLG2 overexpression is linked to a poor prognosis in many cancers, such as prostate, kidney, gastric and triple-negative breast cancer [69,70,71,72]. Although the role of SUCLG2 overexpression is present in many cancers, its potential association with drug resistance is still unclear. In DLKP subpopulations, an increase at the expression levels of SUCLG2 from 1.3 to 1.9-fold was observed with increasing resistance levels.

X-ray Repair Cross-Complementing 5 (XRCC5) is a protein involved in DNA repair. It binds to DNA double strand break ends and is required for the non-homologous end joining pathway. Overexpression of XRCC5 has been implicated in drug resistance in many cancer types, including: temozolomide resistance in glioblastoma, cisplatin resistance in neuroblastoma, and doxorubicin resistance in bone-related cancers [73,74,75]. This study shows an increase at the expression levels of XRCC5 in DLKP cells correlated with increasing resistance. 

Finally, the list of biomarkers which were identified to have significant changes upon drug resistance formation were investigated for their presence in various lung cancer cells and their drug resistant counterparts (Table 3). For this comparison DE data obtained from SK-MES-1 (Lung squamous cell carcinoma) and its Adr and taxotere resistant subpopulations as well as Non-Small Cell Lung Cancer (COR23) and its Adr resistant subpopulations were used. Four of these proteins: ACACA, SRI, DIAPH1 and CLIP1 were observed to be expressed in all lung cancer types, whereas 15 of them were found to be shared by at least 2 different lung cancer types. 

**Figure 7:**
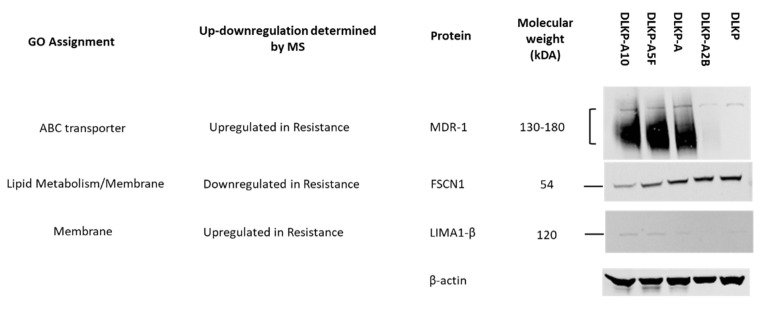
Western blotting of some of the target proteins (MDR1, FSCN1 and LIMA1) and comparison of abundance levels between MS and western-blotting. Full Western blot images of antibodies are provided in Supplementary Figure S5.

Some of the proteins which shows significant changes in drug resistance cells identified by MS are validated using western-blotting. MS results originates from changes of abundances of proteins at peptide level (Figure 7). In protein level, these changes were observed to be consistent. MDR-1 (ABCB1), which plays important role in the efflux of chemotherapeutic agents from cells, are observed to be highly upregulated in drug resistant subpopulations. A membrane protein, FSCN1, which plays an important role in lipid metabolism is observed to be significantly downregulated in drug resistant cells. Similar to results obtained by MS, highest abundance of this protein observed in DLKP cells, whereas lowest is detected for DLKP-A10. Another membrane protein LIMA1, which plays a role in cholesterol metabolism is determined to be upregulated in drug resistant cells, showing correlation with MS results.

## 4. Conclusions

In-depth proteomic analysis of lung cancer cells with various resistance levels provides invaluable insights to cellular mechanism leading to variation in drug response. The 35 potential biomarkers were identified in DLKP cells with significant changes in correlation to drug resistance and their functional analysis in terms of proliferation, metastasis and invasion can help in understanding their role in drug resistance. Therefore, it would be of interest to validate the functional impact of the identified biomarkers in drug resistant cells using knock-downs and/or over expressions, in future experiments.

## Figures and Tables

**Table 1:** Drug metabolism related enriched biological processes and molecular functions.

Term ID	Term Description	Observed Gene Count	Background Gene Count	False Discovery Rate
**Enriched Biological Processes**				
GO:0017144	drug metabolic process	112	622	8.45 × 10^−18^
GO:0042493	response to drug	100	900	1.17 × 10^−5^
GO:2001038	regulation of cellular response to drug	9	28	0.0035
GO:2001024	negative regulation of response to drug	8	29	0.0144
GO:0035690	cellular response to drug	33	310	0.0455
**Enriched Molecular Functions**				
GO:0008144	drug binding	226	1710	7.03 × 10^−21^

**Table 2:** Drug resistance related DE proteins which positively (upregulated-green) and negatively (downregulated-red) correlates with drug resistance levels of DLKP subpopulations. pDLKP expression of protein (*n*) = 1.

Accession	Description	Anova (*p*)	A2B	A	A5F	A10
**Differentially expressed proteins in drug related biological processes**						
P09601	HMOX1	0.000186	−3.2	−7.9	−34.5	−56.1
P34913	EPHX2	0.000329	1.2	2.5	3.9	7.4
Q02818	NUCB1	4.23 × 10^−5^	−1.2	−1.9	−2.0	−2.8
P22695	UQCRC2	6.41 × 10^−5^	2.0	2.3	2.5	2.8
P36957	DLST	8.11 × 10^−6^	1.6	1.8	1.9	2.3
Q96I99	SUCLG2	9.31 × 10^−5^	1.3	1.5	1.5	1.9
P08559	PDHA1	0.001339	1.3	1.5	1.6	1.7
P13073	COX4I1	0.000831	−1.2	−1.5	−1.5	−1.6
P13010	XRCC5	0.000174	1.0	1.3	1.4	1.5
**Differentially expressed proteins in drug related molecular functions**						
P34932	HSPA4	0.000191948	1.3	1.5	1.6	1.9
Q9P2J5	LARS1	0.000250465	1.1	1.3	1.7	1.9
O95757	HSPA4L	3.60 × 10^−5^	1.2	1.4	1.5	1.8
Q08211	DHX9	2.72 × 10^−5^	1.2	1.3	1.4	1.6
P50991	CCT4	0.000158646	1.0	1.3	1.4	1.6
P11216	PYGB	0.004613192	1.1	1.2	1.3	1.7
Q9UHJ6	SHPK	3.91 × 10^−6^	2.0	6.1	6.9	10.2
Q16658	FSCN1	7.52 × 10^−7^	−1.5	−1.5	−3.2	−4.7

**Table 3:** List of biomarkers candidates for drug resistance in lung cancer and their expression profiles in different lung cancer cells. * Highest observed in DLKP variants versus parental DLKP cells.

Biological/Molecular Assignment	Gene Name	DLKP vs. Resistant Max. Fold Change *	SK-MES-1 vs. Resistant Max. Fold Change	COR-23 vs. Resistant Max. Fold Change
Drug transport	ABCB1	+(49.8-fold)	+(13.5-fold)	-
	ABCC1	+(18.8-fold)	-	+(15.1-fold)
Lipid metabolism	ABCD1	−(131.1-fold)	-	-
	ABCD3	+(2.0-fold)	+(4.1-fold)	-
	ALDH1B1	+(2.6-fold)	-	-
	ALDH3A2	+(2.6-fold)	+(4.9-fold)	-
	GCDH	+(4.4-fold)	-	-
	FASN	+(1.6-fold)	-	-
	ACACA	+(2-fold)	+(2.7-fold)	−(2.1-fold)
	CROT	+(52.8-fold)	-	-
Membrane proteins	LIMA1	+(10.7-fold)	-	−(1.5-fold)
	SRI	+(3.1-fold)	+(4.8-fold)	+(1.6-fold)
	ANXA1	+(5.1-fold)	-	+(4.6-fold)
	CAST	+(3.1-fold)	+(2.1-fold)	-
	DIAPH1	+(3.0-fold)	−(1.2-fold)	−(3.3-fold)
	CLIP1	+(3.1-fold)	+(5.2-fold)	+(1.9-fold)
	TMUB1	−(6.3-fold)	-	-
	NEU1	−(5.8-fold)	+(2.8-fold)	-
	FSCN1	−(4.7-fold)	-	−(1.3-fold)
Gene Ontology-drug metabolism related DE proteins	HMOX1	−(56.1-fold)	-	-
	EPHX2	+(7.3-fold)	-	-
	NUCB1	−(2.8-fold)	-	−(1.3-fold)
	UQCRC2	+(2.8-fold)	+(1.2-fold)	-
	DLST	+(2.3-fold)	-	−(1.5-fold)
	SUCLG2	+(1.9-fold)	-	-
	PDHA1	+(1.7-fold)	+(2.2-fold)	-
	COX4I1	−(1.6-fold)	-	+(1.3-fold)
	XRCC5	+(1.5-fold)	-	-
	HSPA4	+(1.9-fold)	-	-
	LARS1	+(1.8-fold)	-	+(1.6-fold)
	HSPA4L	+(1.8-fold)	−(2.1-fold)	-
	DHX9	+(1.6-fold)	-	+(1.5-fold)
	CCT4	+(1.6-fold)	-	+(1.6-fold)
	PYGB	+(1.7-fold)	-	+(1.6-fold)
	SHPK	+(10.3-fold)	-	−(2.1-fold)

## Data Availability

The mass spectrometry proteomics data have been deposited to the ProteomeXchange Consortium (http://proteomecentral.proteomexchange.org) via the PRIDE [76] partner repository with the dataset identifier PXD036996 and 10.6019/PXD036996.

## Supplementary Materials

The following supporting information can be downloaded at: https://www.mdpi.com/article/10.3390/biom12101401/s1, Figure S1: Expression levels of membrane proteins: PTPRC (A) and LRBA (B); Table S1: List of ABC transporters differentially expressed in pDLKP cells and drug-resistant subpopulations. + represents overexpression and − represents downregulation. N/D indicates Not Detected/Not Expressed; Figure S2: Expression levels of Calpain-2 in pDLKP cells and its drug resistant subpopulations; Figure S3: Expression profiles of downregulated membrane proteins in pDLKP and its drug-resistant subpopulations (following different trend for DLKP-A5F); Figure S4: Expression profiles of downregulated membrane proteins in pDLKP and its drug-resistant subpopulations (no correlation with resistance levels); Figure S5: Western Blot images of MDR1, LIMA1 and FSCN1; Supplementary File S1: (A) Significantly enriched KEGG Pathways based on differential expression analysis of pDLKP cells and drug-resistant variant. (B) List of proteins (*n* = 48) involved in fatty acid metabolism and proteasome in addition to ABCD1 and ABCD3; Supplementary File S2: 108 membrane proteins significantly altered between pDLKP cells and its drug-resistant variants; Supplementary File S3: (A) Differential expression analysis of pDLKP cells and drug resistant subpopulations.; (B) Enriched biological processes and metabolic functions related to drug-related mechanisms and list proteins involved in these enrichments. (C) DE proteins which show correlation with drug resistance levels and play important role in the enrichment of drug resistance-related biological processes and molecular functions.
