# Supplementary material for: Label-Free Quantitative Proteomics Analysis of Adriamycin Selected Multidrug Resistant Human Lung Cancer Cells
Source: Biomolecules. 2022 Oct 1;12(10):1401. doi: 10.3390/biom12101401 (PMC9599763; doi:10.3390/biom12101401)
Supplement: Supplementary file 1 [file biomolecules-12-01401-s001.zip › biomolecules-1878417-supplementary_revised300922.pdf]

Label-free Quantitative Proteomics Analysis of Adriamycin selected Multidrug Resistant Human Lung Cancer Cells

Esen Efeoglu[1], Michael Henry[1], Martin Clynes[1,2] and Paula Meleady[1,2,3,*]

[1] National Institute for Cellular Biotechnology, Dublin City University, Glasnevin, D09NR58, Dublin, Ireland
[2] SSPC, The Science Foundation Ireland Research Centre for Pharmaceuticals, V94 T9PX Limerick, Ireland
[3] School of Biotechnology, Dublin City University, Glasnevin, D09 E432, Dublin, Ireland
* Correspondence author: paula.meleady@dcu.ie; Tel.: +353-1-7005910

Supplementary information

Table S1. List of ABC transporters differentially expressed in pDLKP cells and drug-resistant subpopulations. + represents overexpression and - represents downregulation. N/D indicates Not Detected/Not Expressed.

ABC transporters	A2B	A	A5F	A10
**Functional role in Drug transmembrane transport**				
ABCB1 (MDR1)	+1.8	+27.7	+49.8	+34.4
ABCC1 (MRP1)	N/D (Infinity)	+18.8	N/D (Infinity)	+5.8
**Other ABC transporters**				
ABCD1	−131.1	−35.6	N/D (Infinity)	−46.6
ABCF2	−1.4	−1.6	−1.9	−1.6
ABCD3	2	1.2	1.4	1.2
ABCE1	1.6	1.5	2.2	1.5

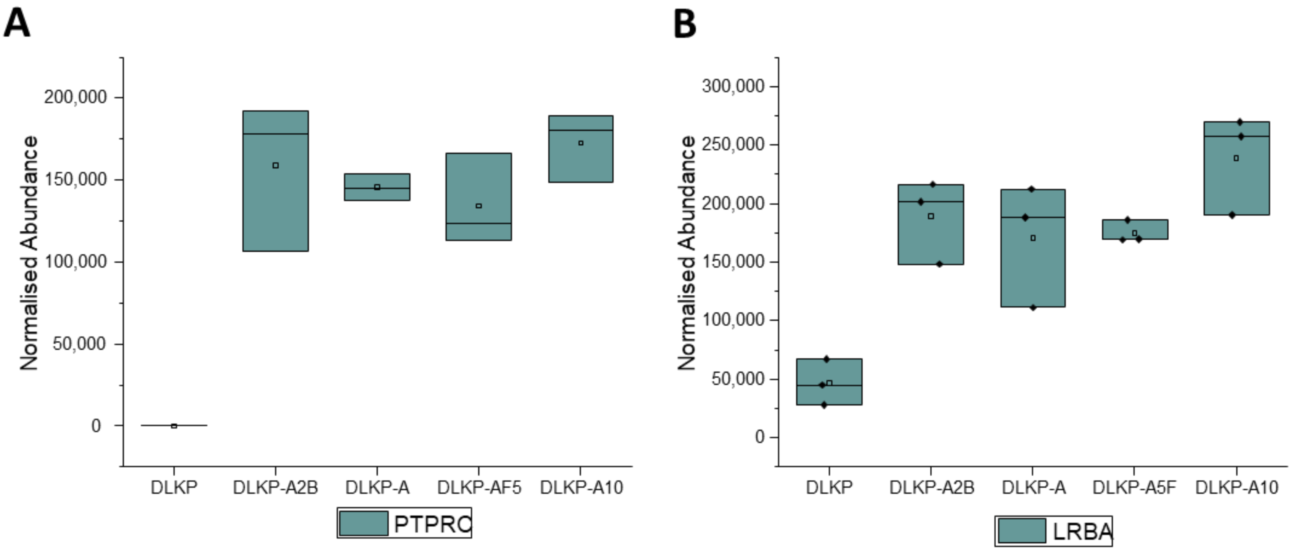

Figure S1. Expression levels of membrane proteins: PTPRC (**A**) and LRBA (**B**).

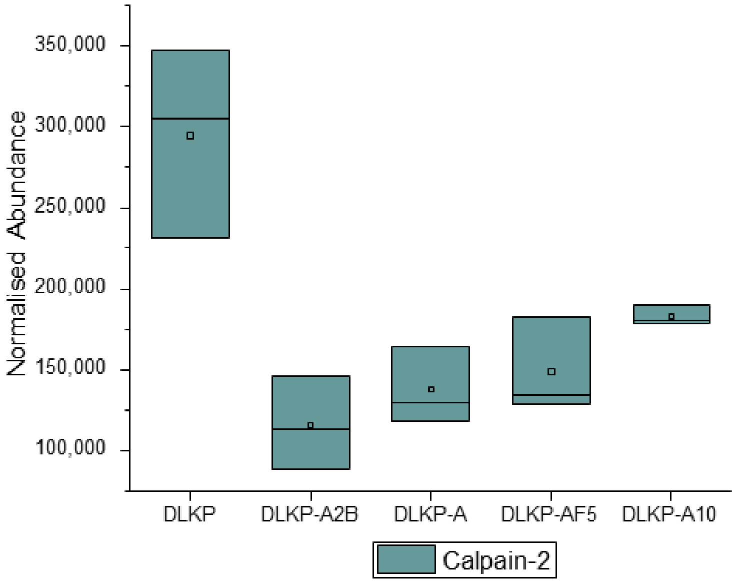

Figure S2. Expression levels of Calpain-2 in pDLKP cells and its drug resistant subpopulations.

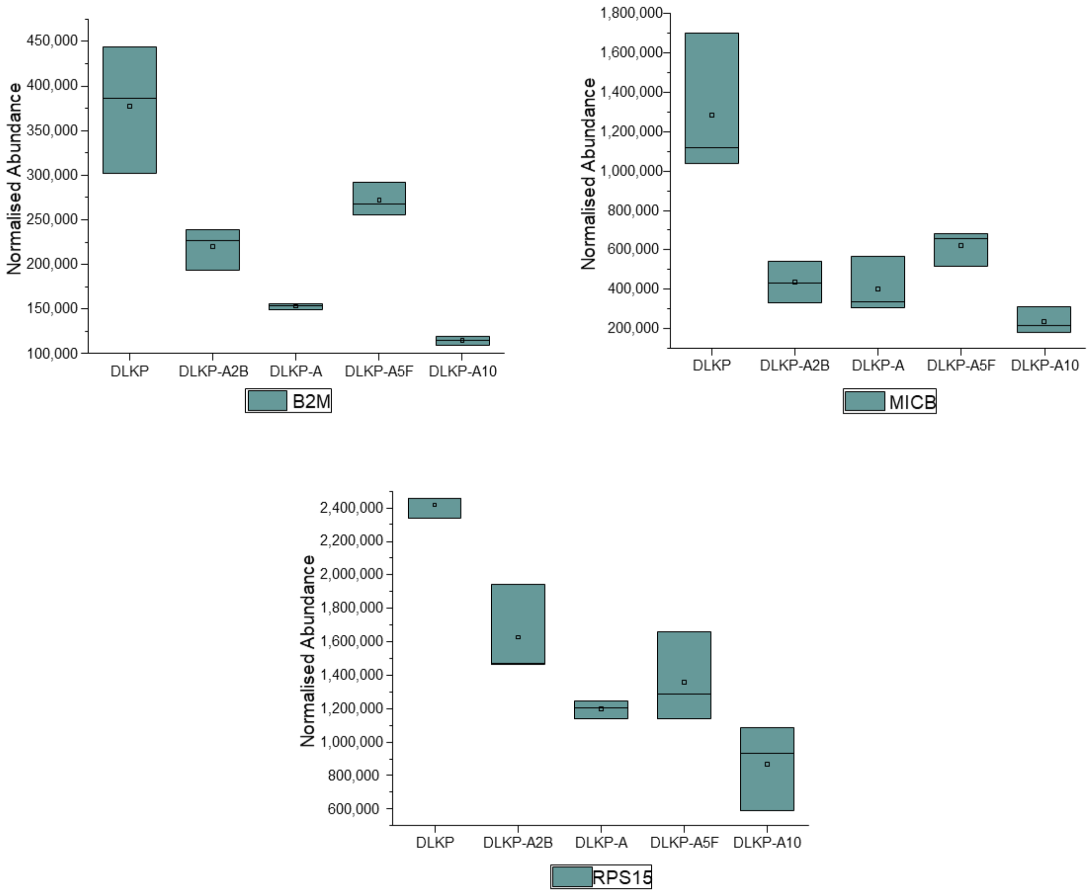

Figure S3. Expression profiles of downregulated membrane proteins in pDLKP and its drug-resistant subpopulations (following different trend for DLKP-A5F).

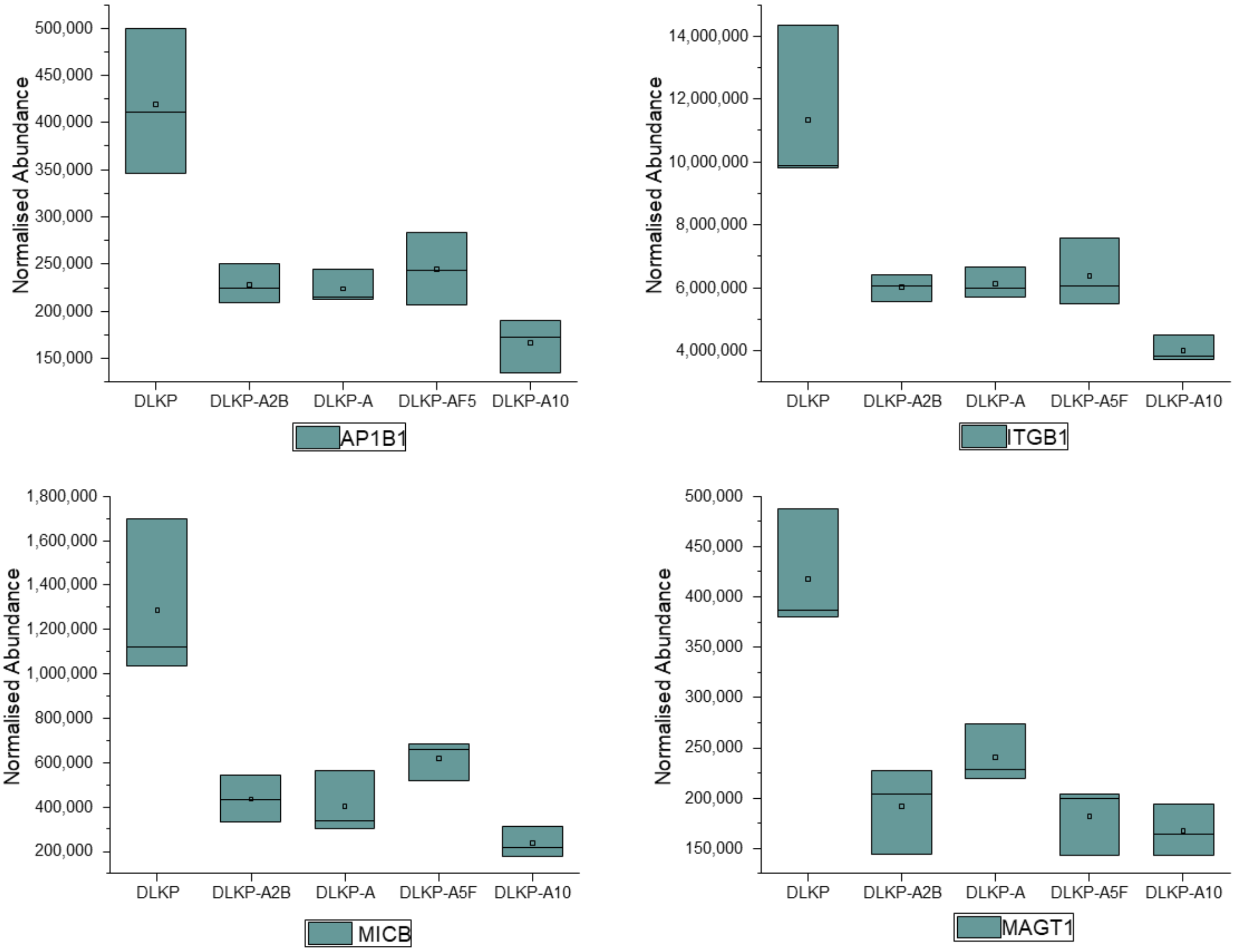

Figure S4. Expression profiles of downregulated membrane proteins in pDLKP and its drug-resistant subpopulations (no correlation with resistance levels).

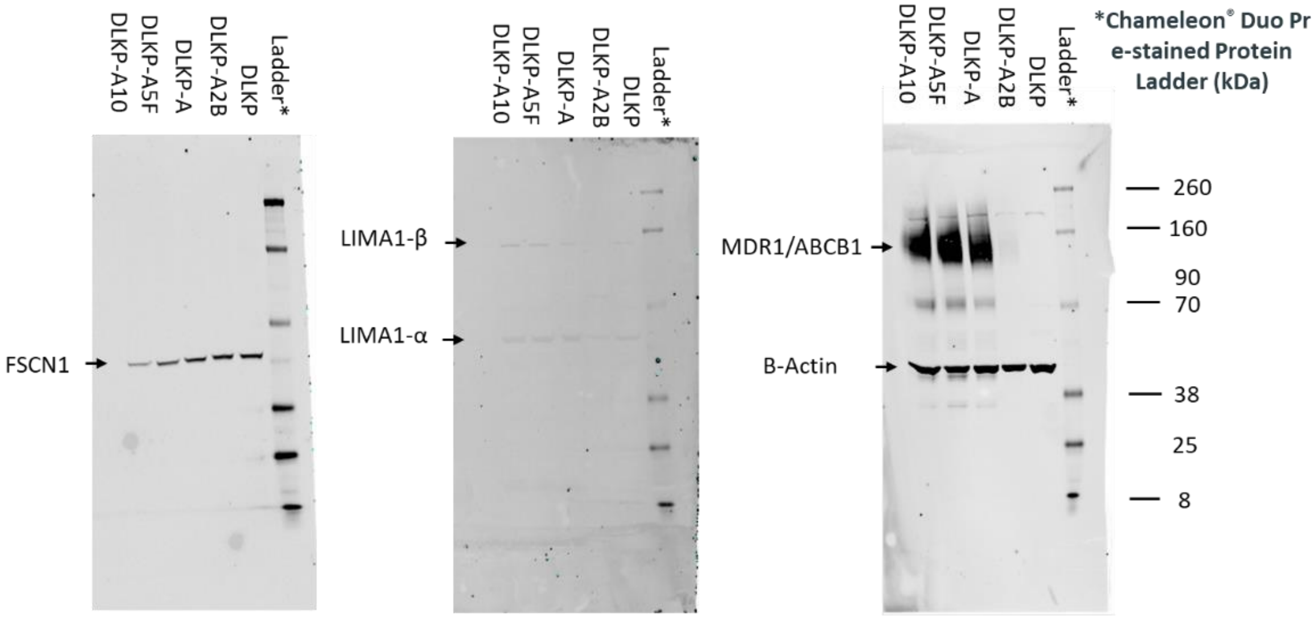

Figure S5. Western Blot images of MDR1, LIMA1 and FSCN1.
